# Comparative Analyses of Rhizosphere Bacteria Along an Elevational Gradient of *Thuja sutchuenensis*

**DOI:** 10.3389/fmicb.2022.881921

**Published:** 2022-05-03

**Authors:** You-wei Zuo, Jia-hui Zhang, Deng-hao Ning, Yu-lian Zeng, Wen-qiao Li, Chang-ying Xia, Huan Zhang, Hong-ping Deng

**Affiliations:** ^1^Center for Biodiversity Conservation and Utilization, School of Life Sciences, Southwest University, Chongqing, China; ^2^Chongqing Key Laboratory of Plant Resource Conservation and Germplasm Innovation, School of Life Sciences, Institute of Resources Botany, Southwest University, Chongqing, China; ^3^Chongqing Academy of Science and Technology, Low Carbon and Ecological Environment Protection Research Center, Chongqing, China

**Keywords:** *Thuja sutchuenensis*, elevational variation, bacterial community diversity, rhizosphere, soil properties

## Abstract

*Thuja sutchuenensis* Franch. is an endangered species in southwestern China, primarily distributed in 800–2,100 m of inaccessible mountainous areas. Rhizosphere soil physicochemical properties and bacterial communities play an essential role in managing plant growth and survival. Nonetheless, the study investigating rhizosphere soil properties and bacterial communities of *T. sutchuenensis* is limited. The present study investigated soil properties, including soil pH, organic matter, water content, nitrogen, phosphorus, and potassium contents, and bacterial communities in nearly all extant *T. sutchuenensis* populations at five elevational gradients. Our results demonstrated that the increase in elevation decreased rhizosphere and bulk soil phosphorus content but increased potassium content. In addition, the elevational gradient was the dominant driver for the community composition differentiation of soil bacterial community. Proteobacteria and Acidobacteria were the dominant bacterial phyla distributed in the rhizosphere and bulk soils. Co-occurrence network analysis identified key genera, including *Bradyrhizobium*, *Acidicapsa*, *Catenulispora*, and *Singulisphaera*, that displayed densely connected interactions with many genera in the rhizosphere soil. The dominant KEGG functional pathways of the rhizosphere bacteria included ABC transporters, butanoate metabolism, and methane metabolism. Further correlation analysis found that soil phosphorus and potassium were the dominant drivers for the diversity of soil bacteria, which were distinctively contributed to the phylum of Planctomycetes and the genera of *Blastopirellula*, *Planctomycetes*, and *Singulisphaera*. Collectively, this comprehensive study generated multi-dimensional perspectives for understanding the soil bacterial community structures of *T. sutchuenensis*, and provided valuable findings for species conservation at large-scale views.

## Introduction

China is one of the countries with abundant plant species and harbors the most extensive relic plant lineages (e.g., *Davidia involucrate*, *Cathaya argyrophylla*, and *Ostrya rehderiana*) (Li, 2008; Liu et al., 2019). Nevertheless, due to factors such as human disturbance and global climate change, many endemic plants in China are endangered (Li et al., 2019; Jiang et al., 2020). *Thuja sutchuenensis* Franch. is an evergreen coniferous tree of Cupressaceae, and was first discovered in 1892 by Paul Guillaume Farges in Chengkou County, Chongqing, China (Tang et al., 2015; Yu et al., 2020). Nonetheless, the tree was identified as “extinct” for more than 100 years and was surprisingly rediscovered in October 1999. Currently, *T. sutchuenensis*, a critically endangered plant, is endemic to the Daba Mountains in Chengkou county and Kaizhou District of Chongqing Municipality at 800–2,100 m (Peng and Wang, 2008; Tang et al., 2015). Previous studies showed that the plant diversity of *T. sutchuenensis* forest was decreased with elevation, and the root biomass and height of *T. sutchuenensis* were lower at high altitudes (Tang et al., 2015; Wang, 2017). Mountainous ecosystems provide a wide range of habitats for plants along an elevational gradient, but environmental factors vary distinctively (Wang et al., 2007). Therefore, exploring the regression conditions and habitat restoration for the *T. sutchuenensis* from the spatial stereoscopic and large-scale perspectives is necessary.

Soil is the substrate for terrestrial plants, providing essential mineral elements and water for plants. Also, the soil is a crucial room in exchanging organic matter (OM) and energy in the soil-microbe-plant ecosystem (Qin and Huang, 2010). Soil OM is able to provide nutrients for plants and plays a critical role in promoting soil aggregate structure, physical and chemical properties, water supply, temperature stability, and ventilation (Ondrasek et al., 2019; Cai et al., 2020). In addition, the growth of plants needs the supplement of certain nutrients, including nitrogen (N), phosphorus (P), potassium (K), and some trace elements from the soil (Wang Y. et al., 2021). Significantly, soil microorganisms affect soil formation, material circulation, and fertility evolution and prove the pivotal bond between soil and plants (Bhatti et al., 2017). It is well-documented that soil bacteria serve as critical drivers for plant growth and survival (Bhatti et al., 2017). Soil bacteria can convert nitrogen into ionic ammonia, providing nutrients for plants (Barney, 2020). Moreover, soil bacteria can decompose insoluble minerals in the soil and rapidly dissolve phosphate, thus promoting plant growth (Majeed et al., 2018). Therefore, the integrated study of soil properties and rhizosphere microorganisms could generate essential and reliable theoretical information for managing and protecting endangered species such as *T. sutchuenensis*.

As a micro-environment of plant roots, the rhizosphere is an important site for soil-root-microorganism interaction (Biddle et al., 2008; Bryant et al., 2008). Rhizosphere microorganisms are composed of many beneficial bacteria, playing profound roles in promoting plant growth and development (Guo et al., 2020). Purposeful conservation of microbial communities is important for the survival and expansion of endangered plants. For instance, the protection of rhizosphere bacteria *Haliangium* and *Candidatus Koribacter* synergistically protected *Scutellaria tsinyunensis*, an endangered species endemic to China (Xu et al., 2021). Some rhizosphere microorganisms (e.g., *Trichoderma*, *Mortierella*, and *Hypocrea*) were also assumed to protect the natural habitat of the first-class endangered plant *Cypripedium japonicum* and promote its reproduction (Gang et al., 2017). Therefore, the investigation of rhizosphere bacterial communities can provide profound understandings for the survival and protection of rare and endangered species.

To investigate the relationship between the rhizosphere bacterial communities and *T. sutchuenensis*, we collected rhizosphere and bulk soil samples in nearly all extant *T. sutchuenensis* populations at five elevational levels. We hypothesized that bacterial structure would be affected due to spatial variations and that different bacterial communities would respond distinctively to soil properties. Detailedly, the primary focuses of this study aim to: (1) unveil how rhizosphere soil properties of *T. sutchuenensis* respond to elevational changes; (2) identify the dominant soil bacterial communities and investigate how elevation reshape rhizosphere bacterial composition; and (3) estimate the relationships between bacterial communities and soil properties.

## Materials and Methods

### Plant Survey and Soil Sampling

To comprehensively identify habitat characteristics and distribution of *T. sutchuenensis*, a field survey was conducted on all known field communities of *T. sutchuenensis*. Previous studies have shown that the elevational distribution interval of *T. sutchuenensis* was between 800 and 2,100 m (Peng and Wang, 2008; Tang et al., 2015). Therefore, in the present study, we considered 300 m as an elevational gradient and eventually selected five gradients (i.e., 700–1,000, 1,000–1,300, 1,300–1,600, 1,600–1,900, and 1,900–2,200 m). Specifically, Jianguangzhan (108.7623°E, 31.7135°N, 980 m), Xiaohanxi (108.7574°E, 31.7240°N, 1073 m), Gaojiayan (108.7799°E, 31.7209°N, 1512), Jiguanziliang (108.7318°E, 31.6469°N, 1658 m), and Shuangping (108.8100°E, 31.7095°N, 2119 m) were used as soil sampling sites in this study (Supplementary Figure 1). In order to investigate the composition and function of the soil bacteria, rhizosphere and bulk soil samples (at a depth of 20–60 cm, each group had six replicates) of *T. sutchuenensis* from the five sampling sites were collected. Specifically, the rhizosphere soil samples of *T. sutchuenensis* were collected, as described by Wang and Zabowski (1998), after shoveling the roots approximately 0.5 m away from the trunk. The corresponding bulk soil samples were collected as control about 4 m away from the trunk. The rhizosphere and bulk soil samples were immediately placed in an insulated container with ice and then transported to the laboratory. After removing debris and roots, the rhizosphere and bulk soil samples were well ground and sieved (<2 mm). A portion of the soil samples was stored at −80°C for subsequent DNA extraction, whereas the remaining soil was used for soil physical and chemical characterization.

### Soil Physicochemical Properties

The experimental detection procedures of soil physicochemical properties were performed as previously reported (Bao, 2000). Soil water content (WC) was measured by drying method; pH was measured by potentiometric method; OM was determined by potassium dichromate and sulfuric acid digestion; total nitrogen content (TN) was determined by perchloric acid-sulfuric acid nitrification kjeldahl method; total phosphorus content (TP) was identified by alkali melt aluminum antimony resistance colorimetry; total potassium content (TK) was identified by acid solution-flame spectrophotometry. In addition, the soil available nitrogen content (AN) was measured by alkali hydrolysis diffusion method, the soil available phosphorus content (AP) was measured by hydrochloric acid-sulfuric acid leaching method, and the available potassium content (AK) was measured by ammonium acetate extraction and flame spectrophotometry method.

### DNA Extraction and High-Throughput Sequencing

We employed QIAamp DNA Mini Kit (Qiagen, Dusseldorf, Germany) to extract microbial DNA for each *T. sutchuenensis* soil sample following the manufactural instructions. We tested the integrity of extracted genomic DNA using 1.2% agarose gel electrophoresis. The purified DNA was used as a template to amplify the target fragment using standard primers 515F (5′-GTGCCAGCMGCCGCGG-3′) and 926R (5′-CCGTCAattCMTTTGAGTTT-3′) for the 16S rDNA V4–V5 region (Biddle et al., 2008). PCR amplification was conducted with the following modifications: 94°C (1 min), 35 cycles of 94°C (30 s), 52°C (30 s), 68°C (30 s), and 6°C (10 min). 1.2% agarose gel electrophoresis was performed to verify the quality of the amplified fragments. The sequencing primers and label sequences were added to both ends of the target fragments. PCR products were pooled in equal density ratios and purified with GeneJET Gel Extraction Kit (Thermo Scientific, Waltham, MA, United States). The purified PCR amplicons products were sequenced on the Illumina MiSeq platform (Illumina Inc., San Diego, United States) at the TinyGene Bio-Tech Co., Ltd. (Shanghai, China). The soil bacterial dataset was deposited in the NCBI Sequence Read Archive (BioSample accession: PRJNA780677).

Raw reads yielded by Miseq sequencing were initially differentiated according to the barcode. The sequence quality was controlled, filtered, and then spliced according to the overlapping relationship. The optimized sequence parameters were set to maxAMBIG = 0, maxHOMOP = 8, MinLength = 200, maxLength = 580. Operational Taxonomic Units (OTUs) indicate the same markers artificially assigned to a taxon (e.g., genus, species, and grouping) for precise analysis in phylogeny or population genetics studies (Blaxter et al., 2005). The high-quality tags processed above were clustered for OTU and classified species annotation using USEARCH software (Edgar, 2010). Quantitative Insights into Microbial Ecology (QIIME) software was performed to generate 16S rRNA OTUs (Caporaso et al., 2012). Finally, a total of 8,025,259 high-quality bacterial sequences and 13,187 OTUs were yielded based on the high-throughput sequencing and processing. Soil bacterial function between rhizosphere and bulk soil samples were enriched using PICRUST2 based on Kyoto Encyclopedia of Genes and Genomes (KEGG) pathway analysis (Douglas et al., 2020).

### Statistical Analyses

Alpha diversity indexes, including Ace index, Chao1 index, Shannon index, and Simpson index, were employed to evaluate species abundance and diversity of bacterial communities. The alpha diversity indexes from five elevations were calculated using Mothur software (Schloss et al., 2009) and analyzed using least-squares regression analysis (Jiao et al., 2018). To investigate the relationship between bacterial community and elevational gradient, we conducted a non-metric multi-dimensional scale analysis (NMDS) of the soil microbial community of *T. sutchuenensis* based on Bray-Curtis distance using “vegan” and “nlme” packages of R program (R Core Team, 2016). The co-occurrence network among different genera was constructed. The Spearman’s correlations between two OTUs were estimated with Spearman’s correlation coefficients *r* > 0.75 and *p* < 0.01 (FDR-corrected) using R packages “igraph” and “Hmisc.” All identified significant correlations from a pairwise comparison of OTU abundance formed a correlation network. To describe network topology, the study calculated a set of metrics (e.g., modularity) and unique node-level topological features (e.g., eigenvector centrality) (Xu et al., 2021). The network was visualized by Gephi software with the layout of Fruchterman Reingold (Jiao et al., 2016). Linear discriminant analysis (LDA) was performed to determine the significant difference of soil bacterial composition in pairwise comparisons (LDA > 3, *p* < 0.05) using R package “lefser” (Clarke et al., 2014).

In addition, we used the two-way ANOVA test to estimate the significance of soil physicochemical properties (i.e., pH, OM, WC, TN, TP, TK, AN, AP, and AK) among different comparisons. The relationships of soil bacterial community with soil properties were calculated using Spearman rank correlation analysis (Clarke et al., 2014). The R package “randomForest” was performed to evaluate the primary drivers of soil taxonomic phyla on soil physicochemical properties (Garge et al., 2013). The contribution of variables was analyzed using the percentage of IncMSE (increased mean square error) (Breiman, 2001). Variations in important soil nutrients with bacterial genera were determined by linear least-squares regression analysis using GraphPad Prism (V.5.02, GraphPad Software, Inc.). The significance levels showing in the study were as follows: *p < 0.05, ^**^p < 0.01, ^***^p < 0.001. Graphics were processed by Adobe Illustrator CC.

## Results

### Soil Physicochemical Properties Along the Elevational Gradient

The results showed that soil physicochemical properties (i.e., WC, TP, TK, AK, pH, AN, and AP) were altered distinctively along the elevational gradient (*p* < 0.05; Supplementary Figure 2). Notably, the contents of rhizosphere and bulk soil WC (*p* < 0.001), TK (*p* < 0.001), and AK (*p* < 0.001) were increased with the elevation and peaked in 2119, whereas the content of TP (*p* < 0.001) and AP (*p* = 0.014) decreased with the elevation. Elevational gradients also altered soil pH (*p* = 0.019) and the content of AN (*p* = 0.007), but did not affect the contents of soil OM (*p* = 0.554) and TN (*p* = 0.271). Pairwise comparison showed that the content of soil TK was increased in rhizosphere compared with bulk soil at 980, 1,512, 1,658, and 2,119 groups (*p* < 0.05; Supplementary Figure 2). Nonetheless, other detected soil properties showed no alterations between rhizosphere and bulk soil (*p* > 0.05).

### Microbial Composition and Classification

**FIGURE 1:**
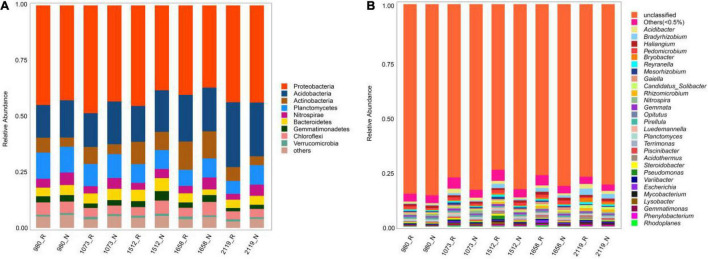
Relative abundance of rhizosphere and non-rhizosphere bacteria across whole elevations. Barplots showing the bacterial community composition at phylum **(A)** and genus **(B)** levels. R and N indicate rhizosphere and bulk soil samples, respectively.

For the rhizosphere and bulk soil bacteria, the majority of bacterial sequences belonged to the phyla Proteobacteria (36.96–48.44%, average: 42.67%), Acidobacteria (14.71–29.15%, average: 19.46%), and Acitinobacteria (3.97–12.70%, average: 7.63%). Compared with bulk soil bacteria, the relative abundances of Proteobacteria in rhizosphere soils at five elevational groups were increased (*p* < 0.05), whereas the relative abundance of Acidobacteria was decreased (*p* < 0.05; Figure 1A). At the genus level, the dominant bacterial communities were *Acidibacter* (0.68–1.91%, average: 1.16%), *Bradyrhizobium* (0.24–2.64%, average: 1.12%), and *Haliangium* (0.74–1.23%, average: 0.98%) (Figure 1B). Among them, the relative abundances of *Acidibacter* and *Bradyrhizobium* were higher in the rhizosphere compared with bulk soil at five elevational groups (*p* < 0.05).

Venn plots (Supplementary Figure 3) showed that, with the exception of 980, the number of specific bacterial OTUs in the bulk soil was lower than in the rhizosphere. In 2119, the number of specific bacterial OTUs in the bulk soil and the number of common bacterial OTUs between the rhizosphere and bulk soil was much lower than in the other elevations, while the number of specific bacterial OTUs in the rhizosphere was much higher than in the other elevations. The number of common bacterial OTUs among each elevation in the rhizosphere was 3197, lower than the value of 3,470 in the bulk soil.

### Diversity of Rhizosphere and Bulk Soil Bacterial Community

**FIGURE 2:**
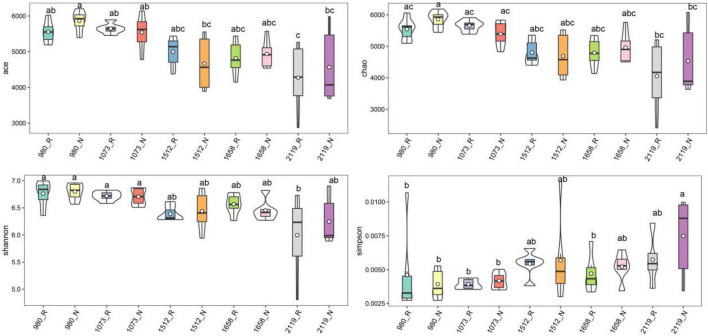
Violin plot showing general patterns of bacterial alpha-diversity along the elevational gradient. The violin frame shows the kernel density of the data distribution. Box-whisker plot inside the violin plot represents the degree of dispersion. ^abc^Different superscripts indicate the significant difference between the pairwise comparison (*p* < 0.05). R and N indicate rhizosphere and bulk soil samples, respectively.

**FIGURE 3:**
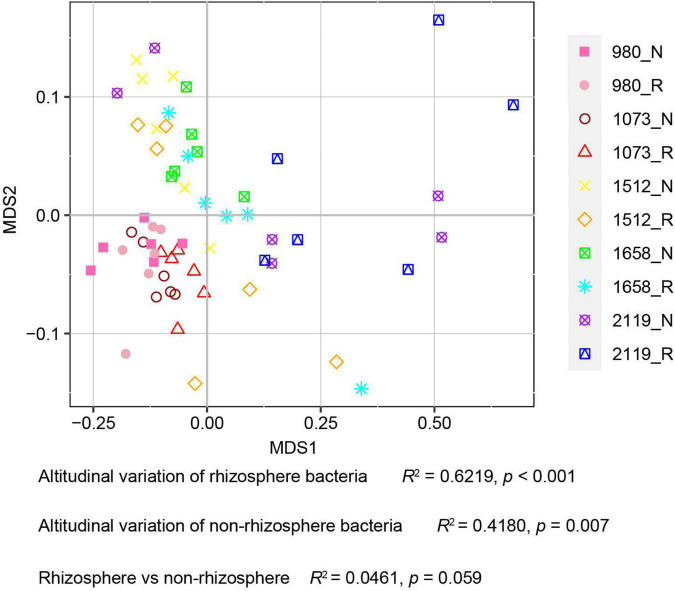
Non-metric multi-dimensional scale analysis (NMDS) analysis for *T. sutchuenensis* soil beta diversity. The beta diversity is calculated based on the relative abundances of OTUs. Values of *R*^2^ and *p* were calculated using Permutational MANOVA (Permanova). R and N indicate rhizosphere and bulk soil samples, respectively.

In this study, alpha diversity indexes (i.e., Chao1, Ace, Shannon, and Simpson) were used to indicate community richness and diversity. The results showed that the bacterial richness and diversity in the rhizosphere and bulk soil were decreased with the elevation (Figure 2). Pairwise comparison showed that alpha diversity indexes in the rhizosphere were not altered compared with the bulk soil. Specifically, Ace, Chao1, and Shannon indexes were higher in the 980 group, whereas the Simpson index was higher in the 2119 group. In addition, we further verified the alteration trend of the alpha diversity indexes based on the least square linear regression analysis (Supplementary Figure 4). Similarly, the results indicated bacterial diversity and richness decreased with elevation (*p* < 0.05). The present study subsequently analyzed the beta diversity to evaluate the similarity and difference of variations to the soil bacterial community. NMDS analysis identified that the *T. sutchuenensis* rhizosphere and bulk soil samples from five elevations represented significant clusters in the ordination space (Figure 3). The results indicated that elevation had a strong impact on the soil bacterial communities in the rhizosphere (*p* < 0.001) and bulk soil (*p* = 0.007). Across the whole elevations, the bacterial communities in the rhizosphere differed slightly (*p* = 0.059) from those in the bulk soil.

### Co-occurrence Analysis of Bacterial Community

**FIGURE 4:**
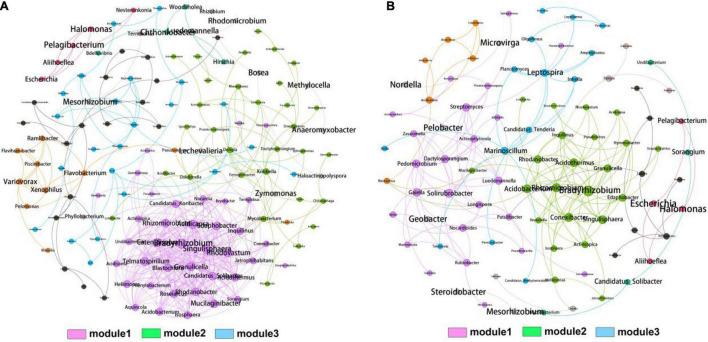
Co-occurrence network analysis of bacterial community from rhizosphere **(A)** and bulk soil **(B)** samples. A connection represents a strong (spearman’s *r* cutoff > 0.75) and significant (*p* cutoff < 0.01) correlation. The size of each node is proportional to the degree of connections. Three MCODE modules with close interaction were identified based on the modularity.

To further explore the role of rhizosphere and bulk soil bacteria across the whole elevations, we constructed network analysis to investigate the co-occurrence patterns in the rhizosphere and bulk soil bacterial genera (Figures 4A,B). The networks consisted of 140 nodes and 416 edges for rhizosphere and 93 nodes and 173 edges for bulk soil, respectively. The modularity indexes in the rhizosphere and bulk soil were 0.472 and 0.688, respectively, indicating the modularization in the generated networks. The two networks could be divided into three distinctive modules after modularized distributions. Notably, module1 harbored the most connected OTUs and the highest density in the rhizosphere and bulk soil. The top four genera, *Bradyrhizobium* (1), *Acidicapsa* (0.949), *Catenulispora* (0.937), and *Singulisphaera* (0.917), were identified as hub biomarkers for rhizosphere due to their high eigenvector centrality scores. The top four genera, *Bradyrhizobium* (1), *Acidothermus* (0.748), *Singulisphaera* (0.724), and *Acidobacterium* (0.711), were identified as hub biomarkers for bulk soil. Statistically, the eigenvector centrality scores of rhizosphere hub genera were higher than bulk soil, indicating that the rhizosphere bacterial community harbored more intense interactions than bulk soil.

### Significant Differences of Rhizosphere and Bulk Soil Bacterial Composition and Function

Linear discriminant analysis was conducted to unveil specific differences in rhizosphere bacterial genera across the whole elevations (Supplementary Figure 5). The results showed that the most number of bacterial genera were detected in 1,073 (LDA > 3), while few taxa were in 2,119. Across the entire elevation, the number of bacterial genera in the bulk soil was much higher than in the rhizosphere. In the rhizosphere, the dominant bacterial indicators were *Variibacter*, *Rhodomicrobium*, and *Rhizobium* from Proteobacteria.

**FIGURE 5:**
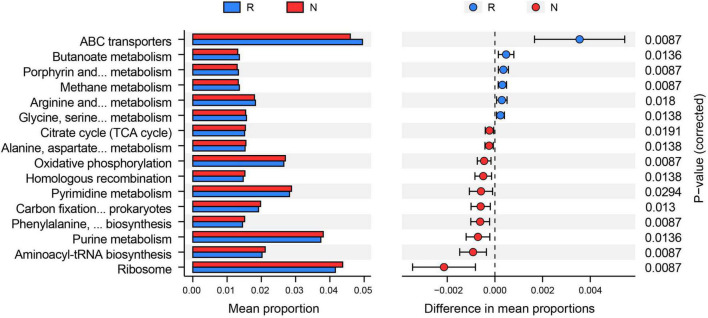
Functions of bacterial community based on Kyoto Encyclopedia of Genes and Genomes (KEGG) analysis. PICRUST2 predicted the alterations of the KEGG pathway between rhizosphere (R) and bulk soil (N) (adjusted *p* < 0.05). The mean proportion of the KEGG function was calculated to indicate relative abundance in the two groups.

We subsequently conducted enrichment analysis of bacterial functions based on the KEGG database to explore different microbial communities’ functional diversity. The results showed that a total of 16 KEGG pathways were found to be distinctively altered by comparing two groups (Figure 5). Among them, six pathways (e.g., ABC transporters, butanoate metabolism, and methane metabolism) were highly enriched in the rhizosphere, whereas the rest of the ten pathways (e.g., ribosome, aminoacyl-tRNA biosynthesis, and purine metabolism) were more abundant in the bulk soil.

### Relationships of Rhizosphere and Bulk Soil Bacteria With Soil Properties

According to Supplementary Figure 2, elevation and rhizosphere had a remarkable impact on soil physicochemical properties, especially soil WC, TP, TK, AP, and AK. Hence, Spearman correlation analysis was conducted on the soil properties and rhizosphere bacterial alpha and beta diversities (Table 1). The results showed that soil WC, TK, and AK were negatively correlated with alpha diversity but positively correlated with beta diversity in the rhizosphere and bulk soil (*p* < 0.05). Besides, soil TP and AP were positively correlated with alpha diversity but negatively correlated with beta diversity in the rhizosphere and bulk soil.

**TABLE 1:** The relationships of bacterial community diversity with soil properties based on Spearman rank correlation analysis.

	Rhizosphere				Bulk soil			
	Chao1	Shannon	MDS1	MDS2	Chao1	Shannon	MDS1	MDS2
pH	0.3087	0.2953	−0.2990	−0.1049	−0.1329	−0.0434	−0.0622	−0.0408
OM	−0.1292	−0.0569	0.1230	−0.0871	−0.1728	−0.1752	0.1512	0.3204
WC	−0.4396*	−0.2572	0.4122*	0.4605*	−0.4119*	−0.4043*	0.2094	0.3138
TN	−0.1475	−0.0526	0.0916	−0.1114	−0.1408	−0.1290	0.05041	0.3285
TP	0.4517*	0.3298	−0.4309*	−0.3471	0.4713**	0.4735**	−0.4475*	−0.4250*
TK	−0.6781***	−0.5553**	0.6488***	0.4317*	−0.5862***	−0.5904***	0.5032**	0.5221**
AN	−0.1614	−0.1101	0.2026	−0.1107	−0.1712	−0.1524	0.0080	0.4131*
AP	0.4010*	0.3337	−0.3744*	−0.3095	0.3899*	0.3647*	−0.3437	−0.2337
AK	−0.4496*	−0.3666*	0.4636**	0.1591	−0.4412*	−0.4558*	0.5361**	0.4618*

*, **, *** indicate that the correlation is significant at P = 0.05, 0.01, and 0.001, respectively.

**FIGURE 6:**
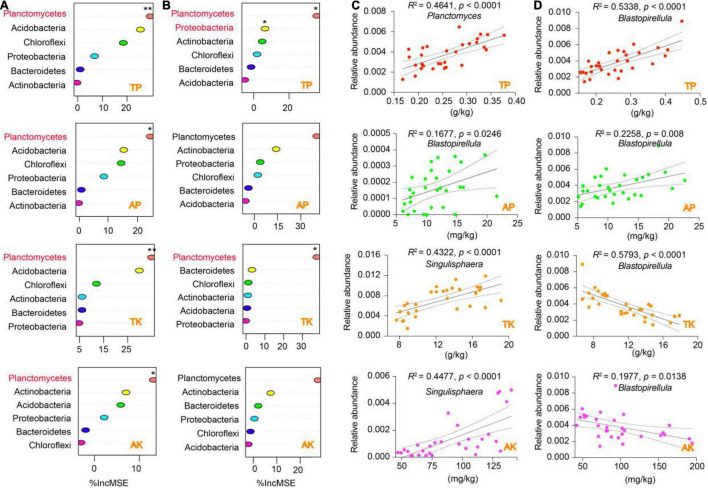
Putative drivers of variation in soil nutrient cycling in the rhizosphere and bulk soil. **(A,B)** Percentage of increase of mean square error (IncMSE) of primary phyla was used to estimate the potential driver of four soil nutrient indicators (i.e., TP, AP, TK, and AK) for rhizosphere **(A)** and bulk soil **(B)**. The accuracy was counted for individual trees and averaged over the whole forest (2,000 trees). Significance levels are as follows: *p < 0.05, **p < 0.01. **(C,D)** Linear regression analysis identified specific bacterial genera explained by soil nutrient indicators for rhizosphere **(C)** and bulk soil **(D)**. The adjusted *R*^2^ and *p* were used to determine the significance of models that were fitted with expectations. The figure also showed the 95% confidence intervals of the best-fit line. Abbreviations: TP, total phosphorus; AP, available phosphorus; TK, total potassium; AK, available potassium.

We performed a random forest (RF) model to identify the specific contributions of the distinctive soil properties (i.e., soil WC, TP, TK, AP, and AK) to bacterial phyla (Figure 6 and Supplementary Figure 6). Interestingly, the results showed that all the soil indicators from the rhizosphere and bulk soil were significantly contributed to the phylum of Planctomycetes (*p* < 0.05), except for soil AP (Figures 6A,B). Also, soil TP significantly contributed to both Planctomycetes and Proteobacteria in the bulk soil (*p* < 0.05). The distinctive contribution of Planctomycetes attracted us to further identify the specific taxa that correlated with the soil indicators. Our regression analysis displayed that three genera from Planctomycetes were significantly correlated with soil WC, TP, AP, TK, and AK. Specifically, for the rhizosphere, *Planctomyces* was positively correlated with soil TP, *Blastopirellula* was positively correlated with soil AP, and *Singulisphaera* was positively correlated with soil TK and AK (Figure 6C). For the bulk soil, *Blastopirellula* was the primary genus positively correlated with soil TP and AP but negatively correlated with TK and AK (Figure 6D). *Planctomyces* was also negatively correlated with soil WC in the rhizosphere and bulk soil (Supplementary Figure 6).

## Discussion

### Soil Properties Were Altered in the Rhizosphere Along Elevation

Soil is the room for plant growth and development, providing essential water, fertilizer, and temperature for plants (Wang et al., 2019; Gong et al., 2020). Specifically, a large number of plants have specific requirements on soil pH and WC. Our results showed that the pH values presented the lowest in the high elevational gradient (2119), and the soil WC also increased with the elevation. The increase of soil WC can provide adequate water in the high-elevation barren soil so that the conifer can perform regular physiological activities based on the well-developed roots dominating the soil layer below 20 cm (Tang et al., 2015). In addition, our results were consistent with a previous study that reported the soil moisture content at alpine shrubs (3,677 m, 32.97%) was distinctively higher than that at the evergreen region (2,986 m, 22.29%) in southwest China (Zhu et al., 2020). The contents of TK and AK in *T. sutchuenensis* soil proved similar patterns; that is, they increased with elevation. An investigation of soil properties in mountainous areas in the southwestern United States showed that soil nutrients and water holding capacity increase with elevation (Bryant et al., 2008). The increased TK and AK contents in high-elevation areas are assumed to help *T. sutchuenensis* grow in high-elevation soils, providing requisite nutrients. Nonetheless, high elevations tend to be P deficient considering the presence of the low-temperature conditions and freeze-thaw circulation (Heerwaarden and Aerts, 2010). In the present study, the same climatic conditions exist, which may be the reason for the decline of TP and AP along the elevation. When considering rhizosphere, only the content of soil TK was increased in rhizosphere compared with bulk soil across whole elevations except for 1073. It is well-documented that K can affect the metabolism of plants (Ghadam et al., 2019). In addition, the increased K content in the rhizosphere can induce the development of the thick outer wall of epidermal cells, thus lowering the occurrence of plant diseases (Dordas, 2008; Bian et al., 2020). Hence, the increased TK and AK content in the rhizosphere might be the dominant factor promoting the growth and population expansion of *T. sutchuenensis*.

### Composition and Diversity of Rhizosphere Bacterial Community Were Diverse at Different Elevation

There are thousands of kinds of soil microorganisms globally. These soil microorganisms play an essential role in the biogeochemical cycle of C, N, P, K, and inorganic metal ions in the biosphere and the decomposition and transformation of various organic matters (Hirsch and Mauchline, 2015; Tian et al., 2021). In order to explore the multi-scale characteristics of the soil microbiome of *T. sutchuenensis*, we used 16S rRNA sequencing technology to conduct characterization analysis on the collected 60 *T. sutchuenensis* soil samples. Our results showed that Proteobacteria and Acidobacteria were the dominant phyla identified in all groups. Bacteria or microbial products from Proteobacteria and Acidobacteria have been marketed to promote plant growth. The beneficial effects of these rhizobacteria on plants include biofertilization, stimulation of root growth, rhizoremediation, and antibiosis (Lugtenberg and Kamilova, 2009). In addition, the co-occurrence network displayed that the most connected module (module1) harbored more OTUs involved in Proteobacteria, Acidobacteria, and Planctomycetes. Based on the eigenvector centrality scores, we identified four nodes (i.e., *Bradyrhizobium*, *Acidicapsa*, *Catenulispora*, and *Singulisphaera*) as the hub genera in the rhizosphere, indicating that these genera might contribute significantly to the composition and diversity of bacterial communities. Notably, the genus *Singulisphaera* (Planctomycetes) plays an important role in soil nutrients (e.g., N, P, and K) degradation and anaerobic oxidation of ammonium (Ivanova et al., 2017). Hence, the four taxa might have critical functions in reshaping the bacterial community in the rhizosphere, and future studies could cultivate them to demonstrate their contribution to the growth of *T. sutchuenensis*. Also, we found that the number of specific OTUs in the rhizosphere was significantly higher in high elevation (2119), and the number of shared OTUs was higher in low elevation (980), indicating that elevation might reshape bacterial community differentiation.

Further results illustrated that elevation was the dominant factor driving the bacterial community differentiation, manifested as alterations of alpha diversity (Chao1, ACE, Shannon, and Simpson) and beta diversity. It is reasonable to assume that bacterial communities vary with elevational gradients because of significant variations in abiotic conditions (Jiao et al., 2018). Our results were in line with a previous study that reported the abundance and phylogenetic diversity of microbial communities in the Rocky Mountains of Colorado decrease with the elevation (Bryant et al., 2008). Similarly, alpine and subalpine elevation soil microbial diversity decreased with increasing elevation (Margesin et al., 2009). Nevertheless, it is attractive that rhizosphere bacterial community diversity was slightly different from bulk soil. *T. sutchuenensis* lives primarily in inaccessible mountainous areas. Therefore, the conservation of bacterial communities may partially explain the low variability and low population differentiation of *T. sutchuenensis* (Qin et al., 2020). To sum up, elevational variation was the dominant factor altering bacterial diversity and structure, and the activity of *T. sutchuenensis* roots could provide a more stable environment for microorganisms.

### Differences of Rhizosphere Bacterial Taxa and Function in Different Elevation Levels

Due to geographic separation, soil bacterial communities tend to display population stratification, such as elevational gradients, vertical depth, and horizontal distances. Among them, elevational gradients distinctively alter soils’ bacterial composition and diversity by affecting plant and soil properties compared with fine-scale spatial variations (Guo et al., 2020). In the present study, the LDA results showed that the dominant bacterial indicators for rhizosphere were *Variibacter*, *Rhodomicrobium*, and *Rhizobium* from Proteobacteria. Accumulating evidence has revealed that the Proteobacteria phylum plays a vital role in enhanced biological phosphorus removal (Yang et al., 2017; Su et al., 2020), suggesting that the alterations of TP and AP might be induced by the specific changes of Proteobacteria. In addition, *Variibacter*, *Rhodomicrobium*, and *Rhizobium* have widely linked with nitrogen fixation and organic matter decomposition, and play essential roles in nutrient recycling in forest ecosystems (Lladó et al., 2018; Dukunde et al., 2019). Exploring the rhizosphere bacterial function is critical to unveil the interactions between plants and microorganisms. Although some studies focused on microbial communities associated with *Thuja* (Weber et al., 2005; McGregor et al., 2019), the bacterial functions in the *T. sutchuenensis* rhizosphere have never been understood. In the present study, we found that six KEGG pathways were highly enriched in the rhizosphere. Notably, previous studies have shown a strong link between ABC transporters, root exudate composition, and *ex planta* (Cox et al., 2019; Wang N. Q. et al., 2021). ABC transporters modulate root exudate diversity, composition, and rhizosphere microbe communities (Wang N. Q. et al., 2021). Hence, the altered ABC transporters pathway indicated strong microbe interaction activities in the *T. sutchuenensis* rhizosphere.

### Relationships of Bacterial Communities With Soil Properties

The soil physicochemical results showed that the soil properties, especially soil WC, P, and K content, were altered by elevation, which would inevitably affect the abundance and diversity of rhizosphere bacterial communities. Expectedly, the Spearman correlation analysis showed that the abundance and diversity of rhizosphere and bulk soil were distinctively correlated with soil WC, TP, AP, TK, and AK. Interestingly, the soil properties were positively correlated with alpha diversity indexes (Chao1 and Shannon) but negatively with beta diversity indexes (MDS1 and MDS2). Our findings were consistent with a previous report that demonstrated that soil and root properties (e.g., C, N, and P) were positively correlated with the Shannon index but negatively correlated with the MND1 index (Guo et al., 2020). This correlation probably arises because alterations in nutrient availability, due to differing return rates and soil nutrient cycling along the climate and elevational gradient, ultimately lead to differences in the rhizosphere bacterial community diversity (Jacob et al., 2009). Further analysis demonstrated that the genera *Blastopirellula*, *Planctomyces*, and *Singulisphaera* from Planctomycetes were the dominant contributors to the soil indicators. Growing research has demonstrated that a high moisture content decreases decomposition rates, and soil WC lowers microbial activity by altering diffusion of soluble substrates, bacterial movement, and intracellular water potential (Killham et al., 1993; Schjnning et al., 2003). Therefore, soil moisture content might determine Planctomycetes composition by regulating nutrient availability and cell movement, consistent with a previous study documented by Zhou et al. (2002), who described that soil WC connecting soil particles affects soil bacterial diversity patterns. Also, our correlation analysis was in agreement with a previous report that demonstrated *Blastopirellula* was positively correlated with the accumulation of phosphorus content in sponges, and can improve the growth and pathogen resistance of marine sponges (Ou et al., 2019). In addition, our results showed that soil TP content was also contributed to the phylum Proteobacteria. Proteobacteria phylum plays a vital role in enhanced biological phosphorus removal (Yang et al., 2017; Su et al., 2020), suggesting that the contents of TP decreased with the elevation might be induced by the specific changes of Proteobacteria. Interestingly, *Singulisphaera*, one of the bioindicators identified based on the co-occurrence analysis, was the dominant genus that could explain the alterations of soil TK and AK. Members of the genus *Singulisphaera* are ubiquitous in terrestrial and aquatic environments with diverse conditions. The predominant Planctomycetes populations, such as *Singulisphaera* and *Paludisphaera*, are psychrotolerant bacteria capable of growth at low temperatures and can participate in the degradation of lichen debris and soil nutrients cycling (Kulichevskaya et al., 2012; Ivanova et al., 2016). Collectively, most biomarkers in the rhizosphere and bulk soil of *T. sutchuenensis* were beneficial for plants, and therefore, we could partially speculate that the root activity of *T. sutchuenensis* distinctively promoted the environmental conditions and elevated the beneficial microorganisms.

To further understand the role of soil microorganisms in the survival and expansion of *T. sutchuenensis*, it would be undoubtedly to compare its rhizosphere and bulk soil microbial community in more native and non-native habitats in future research. In addition, to better explore the abiotic and biotic responses of the rhizosphere and bulk soil of *T. sutchuenensis*, investigations of other microorganisms (e.g., specific functional bacteria, arbuscular mycorrhizal fungi, ectomycorrhizal fungi, and azotobacteria) should include in our coming studies.

## Conclusion

In the present study, we collected a total of 60 rhizosphere and bulk soil samples from five elevations to evaluate the soil bacterial community differentiation patterns of *T. sutchuenensis*. Our findings demonstrated that elevational gradient was the primary driving factor for the richness and diversity of soil bacterial community. In addition, the study identified that *Bradyrhizobium*, *Acidicapsa*, *Catenulispora*, and *Singulisphaera* might play a critical role in the nutrient cycling of *T. sutchuenensis*. In conclusion, this integrated study uncovered the *T. sutchuenensis* soil bacterial community differentiation patterns from multi-spatial perspectives, and the identified bacterial communities could generate profound meanings for understanding *T. sutchuenensis* conservation.

## Data Availability Statement

The datasets presented in this study can be found in online repositories. The names of the repository/repositories and accession number(s) can be found below: https://www.ncbi.nlm.nih.gov/genbank/, PRJNA780677.

## Author Contributions

Y-WZ, H-PD conceived and designed the study. J-HZ, C-YX, and HZ helped with the experiment design. Y-WZ, D-HN, Y-LZ and W-QL collected the samples. Y-WZ and D-HN analyzed the data and prepared the figures and table. H-PD and J-HZ helped with the improvement of the manuscript. All authors read and approved the final manuscript.

## Conflict of Interest

The authors declare that the research was conducted in the absence of any commercial or financial relationships that could be construed as a potential conflict of interest.

## Publisher’s Note

All claims expressed in this article are solely those of the authors and do not necessarily represent those of their affiliated organizations, or those of the publisher, the editors and the reviewers. Any product that may be evaluated in this article, or claim that may be made by its manufacturer, is not guaranteed or endorsed by the publisher.

## Abbreviations

OM: organic matter
N: nitrogen
P: phosphorus
K: potassium
WC: water content
TN: total nitrogen
TP: total phosphorus
TK: total potassium
AN: available nitrogen
AP: available phosphorus
AK: available potassium
OTUs: operational taxonomic units
QIIME: quantitative insights into microbial ecology
KEGG: Kyoto Encyclopedia of Genes and Genomes
LME: linear mixed-effects
NMDS: non-metric multi-dimensional scale analysis
LDA: linear discriminant analysis
IncMSE: increased mean square error.
