# Supplementary material for: Comparative Analyses of Rhizosphere Bacteria Along an Elevational Gradient of Thuja sutchuenensis
Source: Front Microbiol. 2022 May 3;13:881921. doi: 10.3389/fmicb.2022.881921 (PMC9111514; doi:10.3389/fmicb.2022.881921)
Supplement: Supplementary file 1 [file Data_Sheet_1.docx]

**Comparative analyses of rhizosphere bacteria along an elevational gradient of *Thuja sutchuenensis***

You-wei Zuo^1,2^, Jia-hui Zhang^1,2^, Deng-hao Ning^1,2^, Yu-lian Zeng^1,2^, Wen-qiao Li^1,2^, Chang-ying Xia^1,2^, Huan Zhang^1,2^, Hong-ping Deng^1,2,3*^

^1^ Center for Biodiversity Conservation and Utilization, School of Life Sciences, Southwest University, 400715, Beibei, Chongqing, China

^2^ Chongqing Key Laboratory of Plant Resource Conservation and Germplasm Innovation, Institute of Resources Botany, School of Life Sciences, Southwest University, 400715, Beibei, Chongqing, China

^3^ Chongqing Academy of Science and Technology, Low Carbon and Ecological Environment Protection Research Center, 401123, Liangjiang New Area, Chongqing, China

^*^ Corresponding author

Hong-ping Deng; E-mail: denghp@swu.edu.cn; Phone number: +86 13883395687


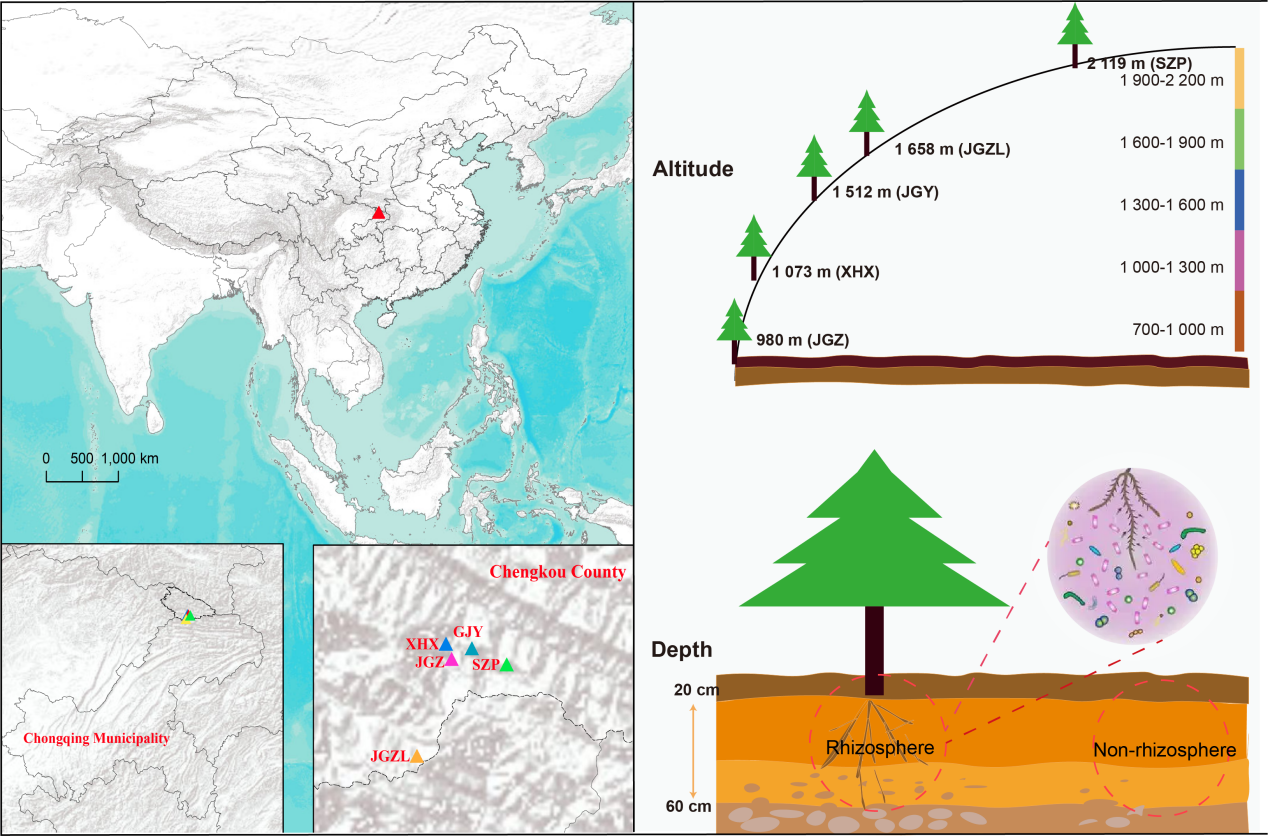


**Fig. S1. Overview of studies comprising the *T. sutchuenensis* rhizosphere and bulk soil sampling sites along the elevational gradient.**


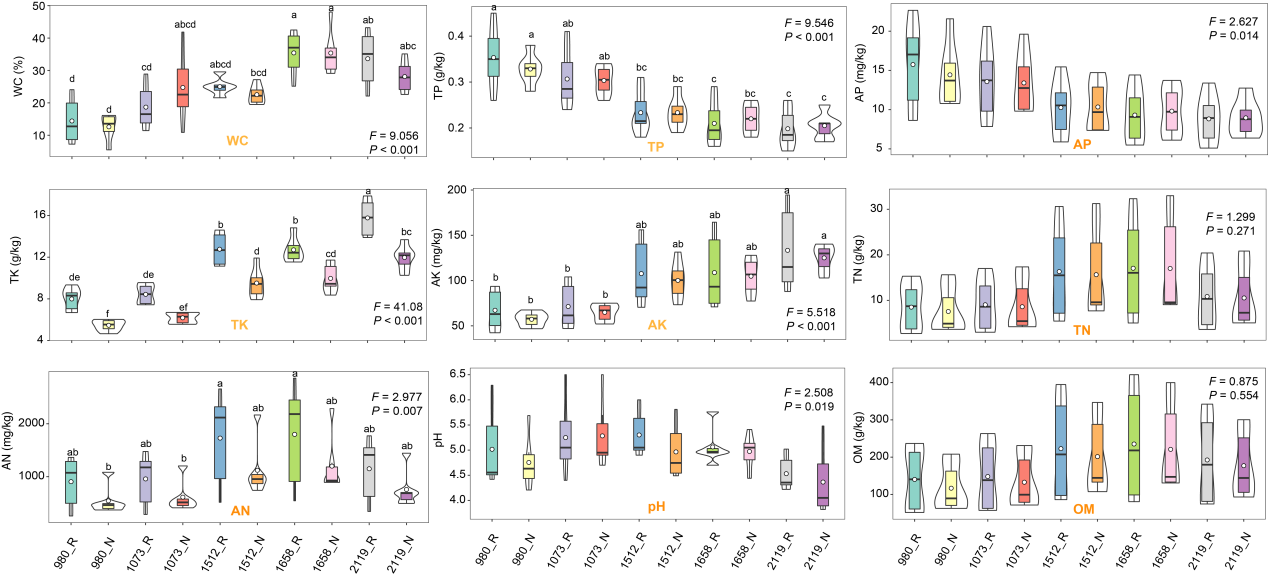


**Fig. S2. Violin plot showing the soil physicochemical properties along the altitudinal gradient.** The violin frame shows the kernel density of the data distribution. Box-whisker plot inside the violin plot represents the degree of dispersion. Abbreviations: organic matter (OM); total nitrogen (TN); available nitrogen (AN); available phosphorus (AP; water content (WC); total phosphorus (TP); total potassium (TK); available potassium (AK); rhizosphere (R); bulk soil (N). *F* and *p* represent the results from ANOVA test across whole elevations. ^abcdef^ Different superscripts indicate the significant difference between the pairwise comparison (*p* < 0.05).


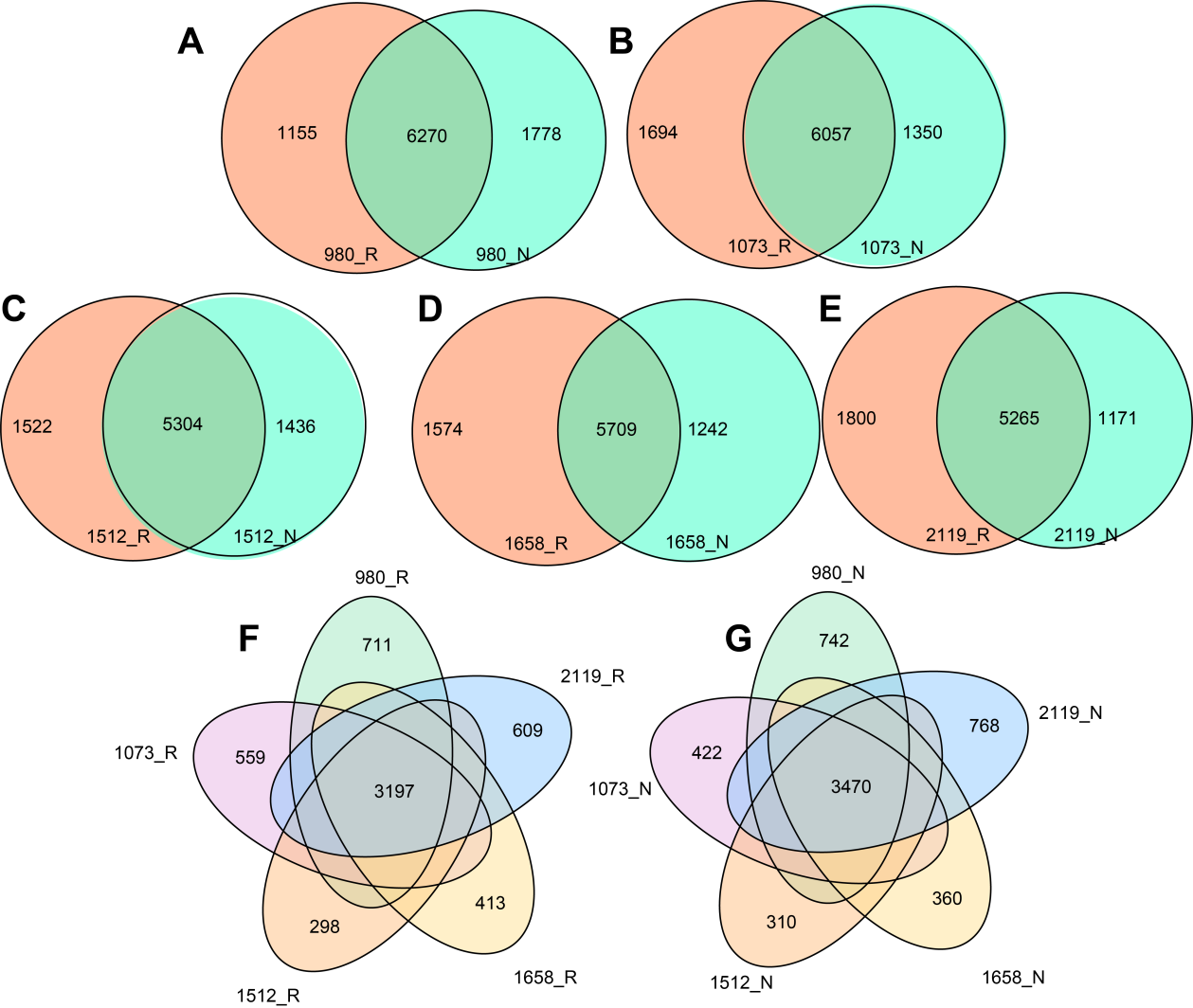


**Fig. S3. Venn plots showing the number of bacterial operational taxonomic units (OTUs) across whole elevations.** (A~E) Specific and shared OTUs at 980, 1073, 1512, 1658, and 2119 groups between rhizosphere and bulk soil samples. (F & G) Specific and shared OTUs in the rhizosphere (F) and bulk soil (G) across whole elevations, respectively. R and N indicate rhizosphere and bulk soil samples, respectively.


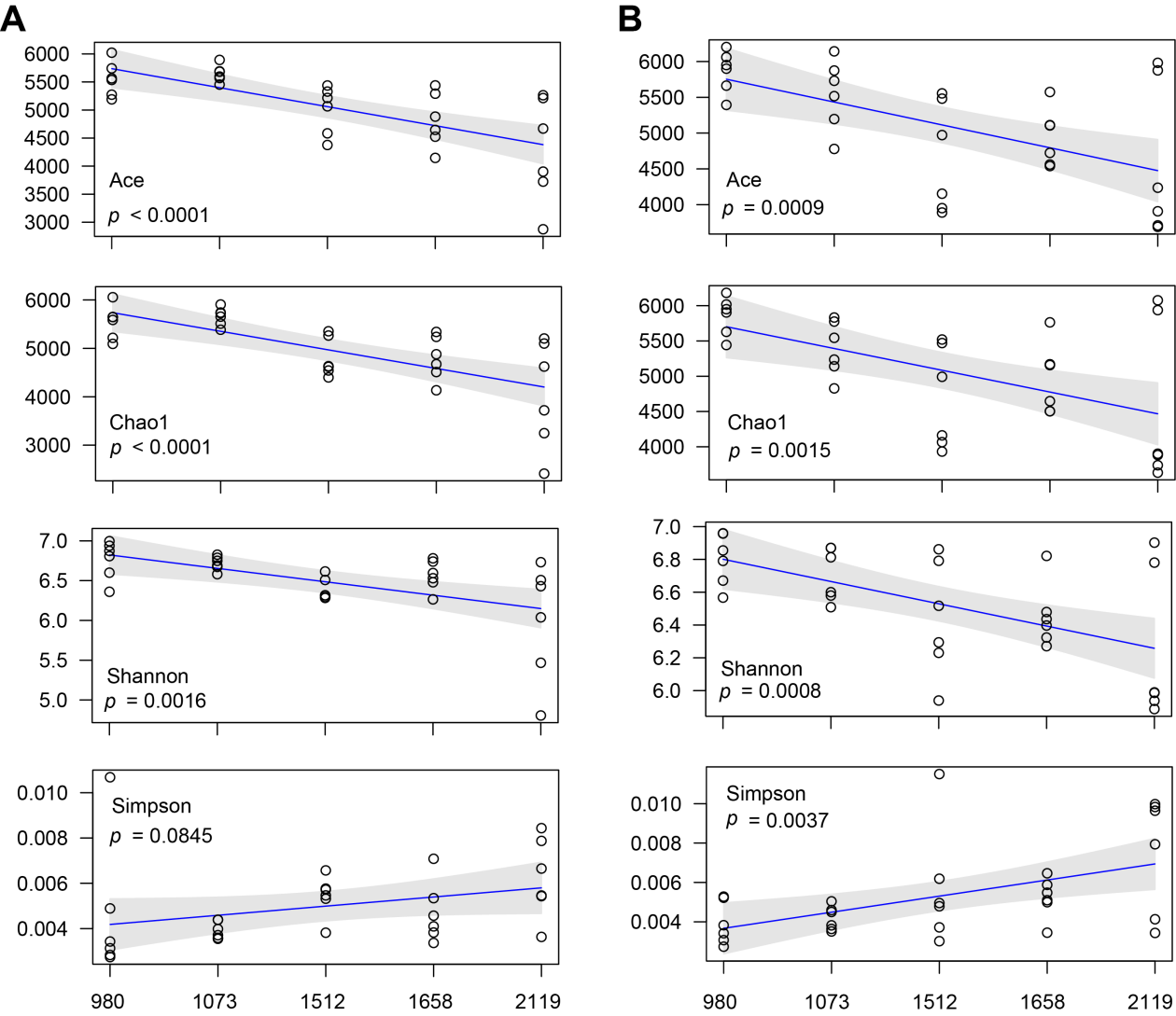


**Fig. S4. The bacterial alpha diversity along the altitudinal gradient. (A)** Rhizosphere; **(B)** Bulk soil. *p* value represents the results from ANOVA test across whole elevations.


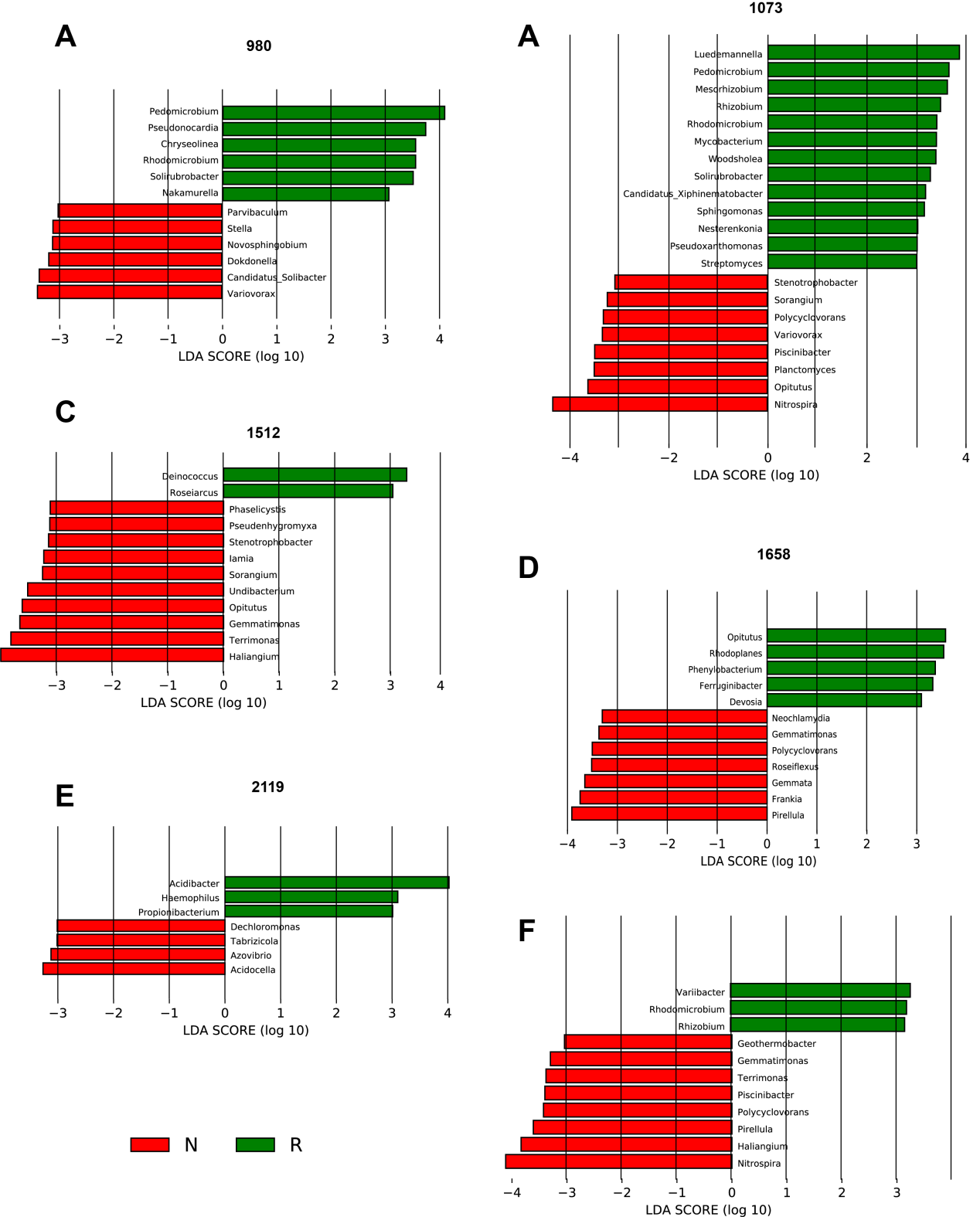


**Fig. S5. The composition of rhizosphere bacterial communities were diverse at different altitudinal gradients.** LDA analysis showed specific differences in rhizosphere bacterial taxa at 980 (A), 1073 (B), 1512 (C), 1658 (D), 2119 (E), and across whole altitudes (980~2119, F). R and N indicate rhizosphere and bulk soil samples, respectively.


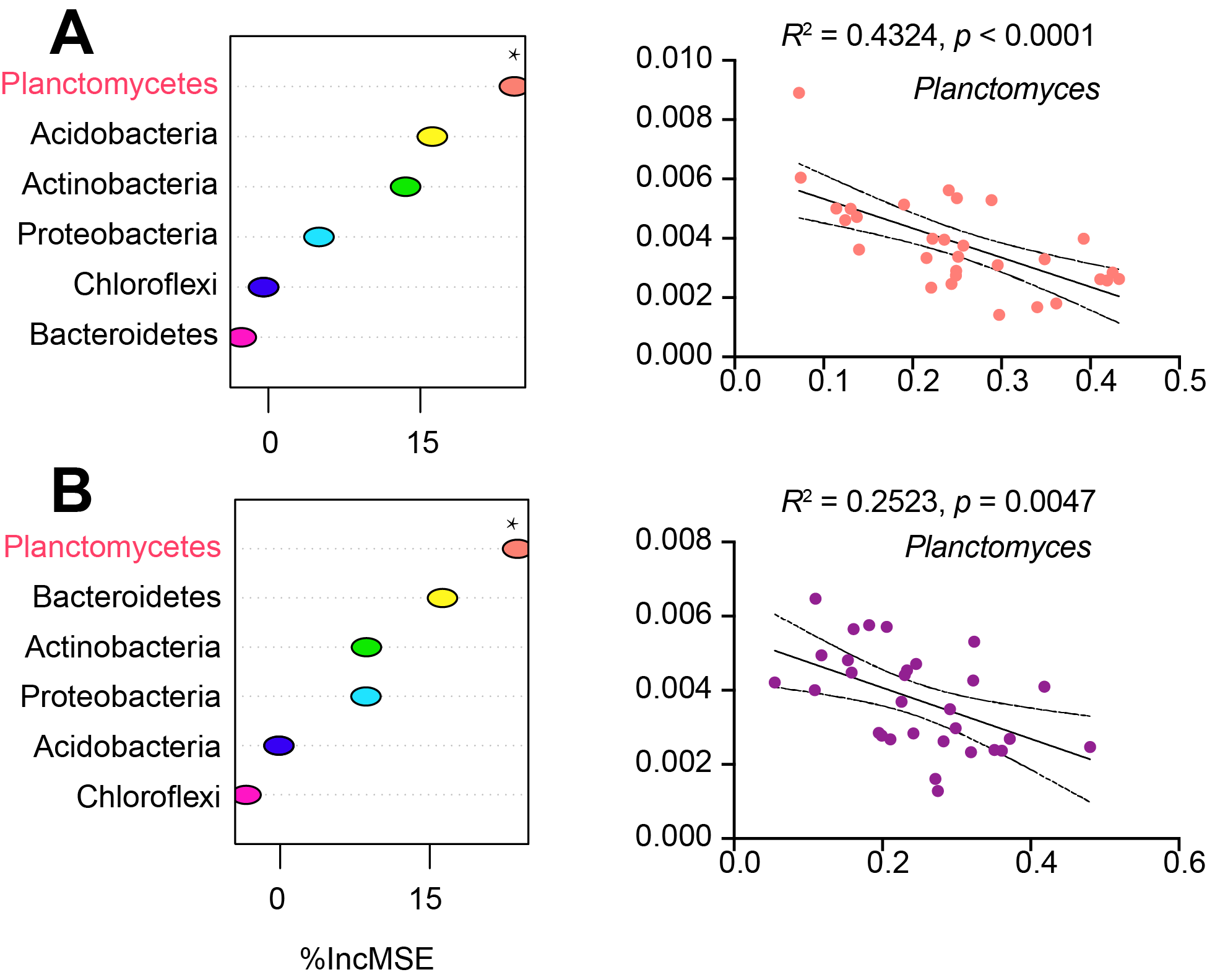


**Fig. S6. Putative drivers of variation in soil water content (WC) in the rhizosphere (A) and bulk soil (B).** (Left panel) Percentage of increase of mean square error (IncMSE) of primary phyla was used to estimate the potential driver of soil WC. The accuracy was counted for individual trees and averaged over the whole forest (2,000 trees). Significance levels are as follows: **p* < 0.05. (Right panel) Linear regression analysis identified specific bacterial genera explained by soil WC.
